# Fertility Among Female Survivors of Childhood, Adolescent, and Young Adult Cancer: Protocol for Two Pan-European Studies (PanCareLIFE)

**DOI:** 10.2196/10824

**Published:** 2018-09-14

**Authors:** Marleen van den Berg, Marloes van Dijk, Julianne Byrne, Helen Campbell, Claire Berger, Anja Borgmann-Staudt, Gabriele Calaminus, Uta Dirksen, Jeanette F Winther, Sophie D Fossa, Desiree Grabow, Victoria L Grandage, Marry M van den Heuvel-Eibrink, Melanie Kaiser, Tomas Kepak, Leontien C Kremer, Jarmila Kruseova, Claudia E Kuehni, Cornelis B Lambalk, Flora E van Leeuwen, Alison Leiper, Dalit Modan-Moses, Vera Morsellino, Claudia Spix, Peter Kaatsch, Eline van Dulmen-den Broeder, The PanCareLIFE Consortium

**Affiliations:** 1 Department of Pediatrics Amsterdam UMC, Vrije Universiteit Amsterdam Netherlands; 2 Boyne Research Institute Drogheda Ireland; 3 Centre Hospitalier Universitaire de Saint-Étienne Saint-Étienne France; 4 Division of Oncology and Hematology Department of Pediatrics Charité – Universitätsmedizin Berlin Berlin Germany; 5 University Children's Hospital Bonn, University of Bonn Medical School Bonn Germany; 6 Pediatrics III University Hospital Essen Essen Germany; 7 West German Cancer Centre German Cancer Research Centre Essen Germany; 8 Danish Cancer Society Research Center Copenhagen Denmark; 9 Department of Clinical Medicine, Faculty of Health Aarhus University Hospital Aarhus Denmark; 10 Oslo University Hospital Oslo Norway; 11 German Childhood Cancer Registry Institute for Medical Biometry, Epidemiology and Informatics University Medical Center Mainz Mainz Germany; 12 University College London Hospital London United Kingdom; 13 Princess Máxima Center for Pediatric Oncology Utrecht Netherlands; 14 Erasmus MC University Medical Center Rotterdam Netherlands; 15 International Clinical Research Center of St Anne's University Hospital Brno Brno Czech Republic; 16 Department of Pediatrics Amsterdam UMC, University of Amsterdam Amsterdam Netherlands; 17 Motol Teaching Hospital Prague Czech Republic; 18 Swiss Childhood Cancer Registry, University of Bern Bern Switzerland; 19 Department of Obstetrics and Gynaecology Amsterdam UMC, Vrije Universiteit Amsterdam Netherlands; 20 Netherlands Cancer Institute Amsterdam Netherlands; 21 Great Ormond Street Children’s Hospital London United Kingdom; 22 Edmond and Lily Safra Children’s Hospital Chaim Sheba Medical Center Tel-Aviv Israel; 23 The Sackler Faculty of Medicine Tel-Aviv University Tel-Aviv Israel; 24 Gaslini Children Hospital Genova Italy

**Keywords:** fertility, late effects, childhood cancer, female, cohort study, case-control study

## Abstract

**Background:**

Despite a significant number of studies on female fertility following childhood, adolescent, and young adult (CAYA) cancer, studies establishing precise (dose-related) estimates of treatment-related risks are still scarce. Previous studies have been underpowered, did not include detailed treatment information, or were based on self-report only without any hormonal assessments. More precise assessments of who is at risk for sub- or infertility are needed.

**Objective:**

The objective of our study is to describe the design and methods of 2 studies on female fertility (a cohort study and a nested case-control study) among female survivors of CAYA cancer performed within the European PanCareLIFE project.

**Methods:**

For the cohort study, which aims to evaluate the overall risk of fertility impairment, as well as the risk for specific subgroups of female CAYA cancer survivors, 13 institutions from 9 countries provide data on fertility impairment. Survivors are defined as being fertility impaired if they meet at least one of 8 different criteria based on self-reported and hormonal data. For the nested case-control study, which aims to identify specific treatment-related risk factors associated with fertility impairment in addition to possible dose-response relationships, cases (fertility impaired survivors) are selected from the cohort study and matched to controls (survivors without fertility impairment) on a 1:2 basis.

**Results:**

Of the 10,964 survivors invited for the cohort study, data are available from 6619 survivors, either questionnaire-based only (n=4979), hormonal-based only (n=72), or both (n=1568). For the nested case-control study, a total of 450 cases and 882 controls are identified.

**Conclusions:**

Results of both PanCareLIFE fertility studies will provide detailed insight into the risk of fertility impairment following CAYA cancer and diagnostic- or treatment-related factors associated with an increased risk. This will help clinicians to adequately counsel both girls and young women, who are about to start anticancer treatment, as well as adult female CAYA cancer survivors, concerning future parenthood and to timely refer them for fertility preservation. Ultimately, we aim to empower patients and survivors and improve their quality of life.

**Registered Report Identifier:**

RR1-10.2196/10824

## Introduction

Advances in diagnosis and treatment of childhood cancer have led to major improvements in 10-year survival rate, which now exceeds 80% [1]. As a consequence, the number of childhood cancer survivors has substantially increased and many of them have reached an age at which they consider parenthood. However, compromised reproductive function is an important and frequently encountered late effect of treatment in female cancer survivors with a high impact on quality of life [2-4]. Alkylating chemotherapy and radiotherapy involving the ovaries have been identified as the 2 main risk factors for fertility impairment, and postpubertal treatment seems to be more gonadotoxic than prepubertal treatment [5,6]. In addition, cranial radiotherapy may also impair fertility, and a possible role for nonalkylating agents must be considered [7].

Despite a significant number of studies on female fertility following childhood and adolescent cancer, studies establishing precise (dose-related) estimates of treatment-related risks are scarce. Previous studies have been underpowered [6,8,9], did not include detailed treatment information [10], or were based on self-report only without any clinical validation [11,12]. In addition, the different methods used to assess fertility (questionnaires, hormonal markers, and ultrasound measurements of the reproductive organs [13,14]), make it difficult to compare studies. More precise assessments of who is at risk, either for immediate and persistent infertility or a shorter-than-anticipated reproductive window, are essential to prevent involuntary childlessness, secondary infertility (ie, incomplete family planning), and increased use of artificial reproductive techniques [15]. Assessments should include both established and relatively new clinical markers, for example, evaluation of menstrual and pregnancy history or levels of follicle stimulating hormone (FSH) and anti-Müllerian hormone (AMH). Moreover, large childhood cancer survivor cohorts with detailed treatment and long-term follow-up data on fertility outcomes are needed to disentangle specific treatment-related fertility risks.

We, therefore, initiated the PanCareLIFE project. This pan-European project, originating from the PanCare network, is a European Union funded project (7th Framework Programme, Theme Health), coordinated by the University Medical Center Mainz (Germany), in which investigators from 10 countries provide data from over 15,000 CAYA cancer survivors [16]. The project is divided into 8 work packages (WP1-WP8), each with distinct activities, and addresses 3 research topics (ototoxicity, fertility, and quality of life). PanCareLIFE strives for survivors of childhood, adolescent, and young adult (CAYA) cancer to enjoy the same quality of life and opportunities as their peers who have not had cancer. The aim of this study is to describe the design, methods, and participating cohorts of 2 PanCareLIFE studies in WP3 (led by Amsterdam UMC, Vrije Universiteit, AUMC) on female fertility, a cohort study and a nested case-control study.

## Methods

### The PanCareLIFE Female Fertility Cohort Study

The aim of the female fertility cohort study is to evaluate the overall prevalence of fertility impairment among female CAYA cancer survivors who are at least 5 years past diagnosis and alive at the time of study assessment. Moreover, it aims to assess the prevalence of fertility impairment for specific subgroups of female CAYA cancer survivors based on cancer diagnosis, type of treatment (simple, yes or no, information on chemotherapy, radiotherapy, and surgery), age at treatment, and calendar period of treatment.

In total, 13 institutions from 9 countries (Germany, Czech Republic, Netherlands, Italy, Switzerland, France, United Kingdom, Norway, and Israel) collect cross-sectional data for the PanCareLIFE female fertility cohort study. These institutions, referred to as data providers, provide data from 16 different institutional cohorts in total. Two of these cohorts are registry-based cohorts (VIVE cohort and the Swiss Childhood Cancer Survivor Study cohort), whereas all other cohorts are hospital-based. Some of these data providers have previously collected their data as part of a local fertility study [3,8,17-21], whereas other data providers collect their data specifically during the PanCareLIFE project. All survivors included in the PanCareLIFE female fertility cohort study were treated between 1963 and 2014. However, each of the 16 cohorts encompasses a specific time period of treatment, as identified by the data providers. In addition, although most cohorts included all types of cancer diagnoses, some cohorts only included survivors who were diagnosed with a specific type of cancer (Table 1; see Multimedia Appendix 1 for an expanded version).

#### Study Population

**Figure 1:**
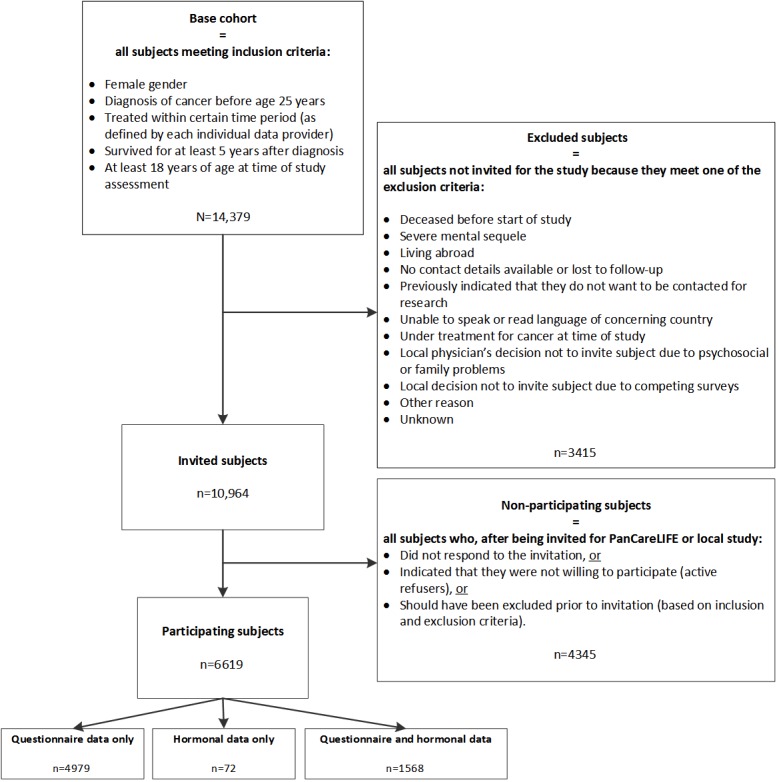
Flow chart of eligible, invited, and participating subjects of the 2 fertility studies within the PanCareLIFE project.

The eligibility criteria for the female fertility cohort study as well as the different survivor groups identified based on eligibility and type of response are described in Figure 1. The base cohort includes all survivors meeting the inclusion criteria. Survivors who subsequently meet one of the exclusion criteria are deemed ineligible and are not invited for the study (excluded subjects). All remaining women have either been invited to participate in a local fertility study in the past or are specifically invited to participate in the PanCareLIFE female fertility study (invited subjects).

Those who do not respond to the invitation as well as those who actively refuse to participate are categorized as nonparticipants. Participants are defined as those who agree to participate by providing either questionnaire data only, hormonal data only, or both. All local ethical committees have approved the use of the collected data from their institute for the PanCareLIFE project.

#### Data Collection

For all women in the base cohort demographic, diagnostic and treatment-related data are collected from medical record files and registries. Basic demographic data include month and year of birth and of latest follow-up. Diagnostic data include type of diagnosis (based on the 3rd version of the International Classification of Childhood Cancer) [22] and month and year of diagnosis. Treatment-related data comprise surgery (yes or no), chemotherapy (yes or no), radiotherapy (yes or no), and bone marrow transplantation (yes or no) complemented with the starting month and year of each treatment. Diagnostic and treatment data are collected for all malignancies and possible relapses.

Data on fertility impairment are collected by questionnaire and hormonal assessments. A specific PanCareLIFE fertility questionnaire is developed for those data providers who collect questionnaire data on fertility issues during the PanCareLIFE project. This questionnaire evaluates sociodemographic and menstrual cycle characteristics, menopausal status, use of oral contraceptives and hormones, reproductive history, and smoking and alcohol behaviors. The questionnaire is translated from the original English into German, Czech, Italian, and Hebrew. All translated questionnaires are back-translated into English (by another translator) to check if the translation is performed properly.

Questionnaire data from questionnaires used by data providers for previous local fertility studies address fertility issues using different questions at different levels of detail and with different answer categories. Therefore, a specific task for WP3 investigators is to recode the relevant data from these questionnaires for compatibility with the variables used in the PanCareLIFE fertility questionnaire in close collaboration with the relevant data provider to make them as compatible as possible.

Hormonal measurements primarily involve the assessment of AMH levels. Study participants are asked to provide a blood sample during a clinic visit (which takes place either as part of standard follow-up care or is specifically scheduled for the study). Part of the sample is centrifuged and stored at −20°C. Subsequently, serum samples are transported in batches by courier to AUMC, where AMH levels are determined centrally in the endocrine laboratory. An ultrasensitive Elecsys AMH assay is used (Roche Diagnostics GmbH, Mannheim, Germany) with an intraassay coefficient of variation of 0.5%-1.8%, a limit of detection of 0.01 µg/L, and a limit of quantitation of 0.03 µg/L [23]. FSH levels are accepted if they have been measured within the previous 2 years or during standard patient care throughout the course of the PanCareLIFE project. FSH measurements are done locally and the results are sent to AUMC. Specifics about the timing of blood sampling, that is, during a natural menstrual cycle, during hormonal contraceptive therapy or hormone replacement therapy, during the pill-free interval, performed anytime (no cycle), during pregnancy, or unknown, are provided.

Data providers collect all data from their own survivor cohort, enter them into a local study database (under a unique PanCareLIFE-ID number), anonymize the data, check the quality of the data, and then send the data to the coordinating PanCareLIFE data center in Mainz. In this center, all subjects are assigned a new unique identification number. Subsequently, the data are compiled and sent to the WP3 investigators at AUMC, as seen in Multimedia Appendix 2.

**Table 1:** Characteristics of cohorts included in the cohort study and the nested case-control study.

Data provider or institute	Study cohort	Data	Women invited (n=10,964) of total base cohort^a^ (N=14,379), n/N	Questionnaires provided (N=6547), n	Serum samples provided (N=1640), n	Time period of data collection
DCOG LATER (Amsterdam UMC, Erasmus Medical Center Rotterdam)^b^, Netherlands	DCOG LATER cohort^c^ [17]	PR^d^	1684/2190	1109	619	2004-2014
Netherlands Cancer Institute Amsterdam, Netherlands	Hodgkin Lymphoma cohort [19,21]	PR	275/450	203	0	1997-2016
Universitätsklinikum Bonn, Germany	VIVE cohort^c^	PR	4467/5909	2482	0	2014-2015
Westfaelische Wilhelms-Universitaet Muenster^b^, Germany	Ewing 2008 Clinical Trials cohort	DU^e^	140/161	46	24	2015-2016
Charité - Universitätsmedizin Berlin, Germany	Berlin Hormone Analyses cohort^c^ [3]	PR	344/402	83	69	2008-2009
Fakultni nemocnice Brno^b^, Czech Republic	Cohort female 5-yr cancer survivors Brno^c^	DU	203/283	182	180	2015-2016
Fakultni nemocnice v Motol^b^, Czech Republic	Cohort female 5-yr cancer survivors Motol^c^	DU	1063/1398	574	301	2014-2016
Istituto Giannina Gaslini^b^, Italy	Gaslini female survivors cohort^c^	DU	814/1111	563	122	2015-2016
University of Bern, Switzerland	Swiss Childhood Cancer Survivor Study cohort 1^c^ [18]	PR	977/1135	685	0	2007-2013
University of Bern, Switzerland	Swiss Childhood Cancer Survivor Study cohort 2^c^ [18]	PR	228/335	113	0	2015-2016
Great Ormond Street Children’s Hospital/University College London Hospital^c^, United Kingdom	Hematopoietic stem cell transplantation cohort^c^	DU	93/95	50	44	2015-2016
Oslo University Hospital^b^, Norway	Lymphoma survivor cohort [8]	PR	82/Unknown	51	46	2007-2009
Oslo University Hospital^b^, Norway	Acute lymphoblastic leukaemia survivor cohort [21]	PR	103/175	82	65	2009-2010
University hospital Saint-Étienne^b^, France	Rhone Alpe cohort 1^c^	PR	120/212	120	35	2005-2013
University hospital Saint-Étienne^b^, France	Rhone Alpe cohort 2^c^	PR	220/284	102	62	2015-2016
Edmond and Lily Safra Children's Hospital, Sheba Medical Center^b^, Israel	The Edmond and Lily Safra Children's Hospital Late Effects cohort^c^	DU	151/239	102	73	2015-2016

^a^Base cohort is the subjects fulfilling inclusion criteria of study.

^b^Institutes participating in the nested case-control study.

^c^Various cancer diagnoses.

^d^PR: data collected prior to PanCareLIFE project.

^e^DU: data collected during PanCareLIFE project.

#### Definition of Primary Outcome

The primary outcome of the cohort study is *fertility impairment*. However, given the fact that the fertility data come from different sources, it is difficult to apply one standardized outcome definition of fertility impairment to all participating cohorts in the cohort study. Therefore, fertility impairment is defined according to 8 criteria based on self-reported and hormonal data (criteria 1, 2, and 6), hormonal data only (criterion 3), or on self-reported data only (criteria 4, 5, 7, and 8). These criteria were established by WP3 with a reproductive specialist (C.B. Lambalk). A survivor is classified as being fertility impaired if she meets at least one of the 8 criteria, as described in Textbox 1.

Low AMH is defined as an AMH level <0.5 µg/L [24]. For criteria 1 through 3, AMH is used to validate self-reported amenorrhea (criteria 1 and 2) or a high FSH level (criterion 3). When AMH levels are used for such validation purposes, levels obtained from serum samples drawn from a participant using any type of hormones are included. However, if only AMH levels are used to decide whether a survivor is fertility impaired or not, like in criterion 6, levels obtained from serum samples drawn during hormonal use are excluded. This is done because previous reports, although inconclusive, have shown that use of contraceptive hormones may significantly decrease AMH levels [25,26]. FSH levels are considered high when they are >30 U/L in a serum sample drawn during the midcycle peak (cycle day 12-16), >15 U/L in a sample drawn at any other moment during the menstrual cycle in case of amenorrhea, or if the survivor uses hormones at time of serum sampling [27]. When there is no information on the timing of the serum sampling, FSH levels are considered high if they are >30 U/L.

**Criteria used to define fertility impairment.**

- Criterion 1: Primary amenorrhea (never had menses) in combination with a high follicle stimulating hormone (FSH) and/or a low anti-Müllerian hormone (AMH) level.

- Criterion 2: Secondary amenorrhea (no menses for >12 months before the age of 40) in combination with a high FSH and/or a low AMH level.

- Criterion 3: High FSH level in combination with a low AMH level, while being <40 years of age at time of study assessment.

- Criterion 4: Primary amenorrhea (without information on AMH or FSH level).

- Criterion 5: Secondary amenorrhea (without information on AMH or FSH level).

- Criterion 6: Low AMH level and <30 years of age at time of study assessment and not using exogenous reproductive hormones at time of blood sampling.

- Criterion 7: Use of artificial reproductive techniques (excluding those who reported male factor as the single cause of subfertility) and being <40 years of age at time of study assessment.

- Criterion 8: Tried to conceive for at least 12 consecutive months without success and being <40 years of age at time of study assessment.

#### Planned Data Analyses

The overall prevalence of fertility impairment will be defined as the number of participating survivors who are fertility impaired divided by the total number of participating survivors. The prevalence of fertility impairment will also be calculated for subgroups based on cancer diagnosis, type of treatment (chemotherapy, +/− surgery; radiotherapy, +/− surgery; both chemo- and radiotherapy, +/− surgery; and surgery only), age group at treatment, and calendar period at treatment. In addition, the prevalence of fertility impairment according to each of the different criteria will be calculated along with that of fertility impairment based on the criteria that evaluate ovarian function (criteria 1 to 6) and possible difficulties getting pregnant (criteria 7 and 8).

Multivariable logistic regression analysis will be used to investigate which diagnostic- or treatment-related risk factors influence the probability of being fertility impaired. All analyses will be adjusted for possible confounders, such as age at the time of study assessment, time since diagnosis, smoking status, BMI, and use of hormonal contraception. Furthermore, to detect possible selection bias, descriptive statistics will be used to describe any differences in age at time of study assessment, age at diagnosis, cancer diagnosis, time since diagnosis, and type of treatment between participants, nonparticipants, and excluded women, as seen in Figure 1. All statistical analyses will be performed by investigators of WP3 (AUMC) in close collaboration with the Biostatistical Support Group of both AUMC and University Medical Center Mainz.

### The PanCareLIFE Female Fertility Nested Case-Control Study

The case-control study is nested within the cohort study, meaning that both cases and controls are selected from participants of the cohort study. However, only participants from institutions that are able to provide detailed treatment data are potential inclusions for the case-control study. This is the case for participants from 11 out of the 16 cohorts in the cohort study (Table 1).

The aims of the nested case-control study are to identify specific treatment-related factors associated with an increased risk of fertility impairment among CAYA cancer survivors and investigate possible dose-response relationships between cumulative dose of radiation from radiotherapy, cumulative dose of specific anticancer drugs, and the risk of fertility impairment.

#### Study Population

The minimal sample size to be included in the nested case-control study population is calculated *a priori*. From a previous study, it was expected that 19% of female childhood cancer survivors exposed to potentially gonadotoxic treatment (ie, the *exposed group*) have low AMH levels compared with 4% among survivors who did not receive such treatment [7]. Based on this information, it is decided to include at least 402 cases and 804 controls (1:2 match), thereby allowing subgroup analyses including up to 6 subgroups (n=67 cases per subgroup). This will enable the detection of an odds ratio of 5.63 with a power of 90% using Fishers’ exact test within the subgroups.

Cases are defined as women who are fertility impaired, as assessed by the 8 criteria described in Textbox 1; controls are defined as survivors without fertility impairment (ie, all women who do not meet any of the 8 criteria). Controls were matched to cases on the following criteria: country of treatment, age at time of study assessment (±1 year), calendar year of treatment (±3 years), and age at first cancer diagnosis (±2 years).

Prior to identifying the cases it is estimated that using these 8 criteria, substantially more than the 402 required cases will be identified. Therefore, to include the 402 cases that are most likely to actually be fertility impaired, the decision was made to hierarchically structure these 8 criteria. Moreover, in making this hierarchy, we also considered the certainty by which each criterion can establish whether the remainder of the participants (ie, the *noncases*) are actually *not* fertility impaired (true controls). Criterion 1 is considered to reflect fertility impairment with the highest certainty and criterion 8 with the least, considering the ability of each criterion to also identify “true controls.” First, women with self-reported (primary or secondary) amenorrhea validated by a high FSH and/or a low AMH level are selected as cases, followed by women with an established high FSH level together with a low AMH level. Subsequently, women who reportedly have amenorrhea (primary or secondary) with no further information on AMH levels are selected, followed by those with an established low AMH level while being younger than 30 years and not using any hormones. Finally, women who indicate by self-report to have ever used some type of artificial reproductive technique and subsequently those who have ever tried to become pregnant for at least one year without success are selected as cases. Ultimately, for the nested case-control study, each case is identified as a case based on one criterion only. The process of case accrual ends after 402 cases are selected.

#### Data Collection

Additional data collected for all selected cases and controls include type, number of cycles, and cumulative doses of each chemotherapeutic agent. For radiotherapy data on site, fractionation schedules and cumulative doses are collected.

#### Planned Data Analyses

Multivariable regression models will be used to investigate which risk factors are most strongly associated with an increased risk of fertility impairment. For this purpose, the associations with individual chemotherapeutic agents and radiotherapy body sites and the risk of fertility impairment will be investigated along with the association with cumulative doses of these chemo- and radiotherapy body sites.

## Results

### The PanCareLIFE Female Fertility Cohort Study

The total base cohort consists of 14,379 female 5-year CAYA cancer survivors, 10,964 of whom are either invited for one of the local fertility studies in the past (n=8500) or for the PanCareLIFE female fertility study (n=2464) (Table 1). In total, data are available from 6619 survivors, either questionnaire-based only (n=4979), hormonal-based only (n=72), or both (n=1568), as seen in Figure 1. Of all questionnaires provided (n=6547), one-quarter (n=1517) consisted of the standardized PanCareLIFE fertility questionnaire. Serum AMH levels have been successfully determined in all 1640 women who provided a blood sample. FSH levels are available from 1242 women.

Table 2 shows the number of women from all participating cohorts who potentially meet each of the criteria for fertility impairment. For some data providers, fertility impairment cannot be assessed by all 8 criteria because not all necessary questionnaire or hormonal data are available for their study population since data were previously collected in local studies.

**Table 2:** Number of participants in the cohort study who could potentially meet the criteria of fertility impairment by participating cohort.

Name of study cohort		Criterion 1 (n=1455)		Criterion 2 (n=1566)		Criterion 3 (n=1207)		Criterion 4 (n=3133)		Criterion 5 (n=5861)		Criterion 6 (n=1640)		Criterion 7 (n=2759)		Criterion 8 (n=5050)	
DCOG LATER cohort	615		615		614		1109		1109		619		1109		0	
Hodgkin Lymphoma cohort	0		0		0		203		203		0		0		0	
VIVE cohort	0		0		0		0		2482		0		0		2482	
Ewing 2008 Clinical Trials cohort	22		22		2		46		46		24		46		46	
Berlin Hormone Analyses cohort	69		69		69		83		83		69		0		0	
Cohort female 5-y cancer survivors Brno	180		180		85		182		182		180		182		182	
Cohort female 5-y cancer survivors Motole	236		236		198		574		574		301		574		574	
Swiss Childhood Cancer Registry cohort 1	0		0		0		0		0		0		0		685	
Swiss Childhood Cancer Registry cohort 2	0		0		0		0		113		0		0		113	
Lymphoma survivor cohort	0		46		36		0		51		46		51		51	
Acute lymphoblastic leukemia survivor cohort	0		65		10		0		82		65		82		82	
Rhone Alpe cohort 1	35		35		9		120		120		35		0		120	
Rhone Alpe cohort 2	62		62		0		101		101		62		0		0	
Gaslini female survivors cohort	122		122		109		563		563		122		563		563	
Hematopoietic stem cell transplantation cohort	42		42		40		50		50		44		50		50	
The Edmond and Lily Safra Children’s Hospital Late Effects cohort	72		72		35		102		102		73		102		102	

**Table 3:** Number of cases and controls identified within study cohorts included in the nested case-control study.

Institute	Study cohort	Cases identified (n=450)	Number of controls matched	
			Controls identified within same cohort (n=801)	Controls identified within DCOG LATER cohort (n=81)
DCOG LATER	DCOG LATER cohort	120	238	N/A^a^
Westfaelische Wilhelms-Universitaet Muenster	Ewing 2008 Clinical Trials cohort	8	15	0
Fakultni Nemocinice Brno	Cohort malignant cancer survivors Brno	17	30	3
Fakultni Nemocnice v Motol	Cohort malignant cancer survivors Motol	128	232	19
Istituto Giannina Gaslini	Gasline female survivors cohort	91	179	2
Great Ormond Street Children’s Hospital and University College London Hospital	Hematopoietic stem cell transplantation cohort	28	6	43
Oslo University Hospital	Lymphoma survivor cohort and Acute lymphoblastic leukemia survivor cohort	18	24	12
University hospital Saint-Étienne	Rhone Alpe cohort 1 and Rhone Alpe cohort 2	28	56	0
Edmond and Lily Safra Children’s Hospital, Sheba Medical Center	The Edmond and Lily Safra Children’s Hospital Late Effects cohort	12	21	2

^a^N/A: Not applicable.

Results show that within the total group of 6619 participants, criterion 1 could be evaluated among 21.98% (1455/6619) of the participants, criterion 2 among 23.66% (1566/6619), criterion 3 among 18.24% (1207/6619), criterion 4 among 47.33% (3133/6619), criterion 5 among 88.55% (5861/6619), criterion 6 among 24.78% (1640/6619), criterion 7 among 41.68% (2759/ 6619), and criterion 8 among 76.30% (5050/6619) of the participants. However, data providers who collect their data during the course of PanCareLIFE collect data for all 8 criteria. This results in a total group of 464 participants for whom all 8 criteria can successfully be evaluated.

All data are collected and entered into local electronic databases by data providers and sent to the coordinating data center in Mainz (WP1). These data are subsequently checked, merged, and cleaned by investigators from WP1 after which a final, aggregated dataset is sent to the investigators of WP3.

### The PanCareLIFE Female Fertility Nested Case-Control Study

The selection of cases and controls has been successfully performed using the hierarchically-ordered criteria of fertility impairment. However, ultimately, it appears that this hierarchy can be discarded since, after the application of the 8th criterion, a total of 504 cases have been identified from the total eligible cohort. Of these, 13 cases are excluded, because no treatment data are available, and 41 because no appropriate matching controls can be found. Therefore, ultimately, 450 cases are included in the case-control study. If the cases identified by the last criterion (criterion 8) are not included in the nested case-cohort study, this will lead to fewer than the required 402 cases.

The 450 selected cases are matched to 882 controls. Some cohort cases cannot be matched to 2 controls owing to an insufficient number of controls in that cohort. However, because the Dutch Childhood Oncology Group - Long term Effects after Childhood Cancer cohort (see Table 1) includes more eligible controls than required, this cohort was used as a “back-up” control selection cohort [17]. In total, 9% (81/882) matched controls are selected from this cohort (Table 3). Overall, 2 matching controls are found for 432 cases, whereas for 18 cases, only one matching control could be identified. After the selection of cases and controls has been finalized, data providers are provided with a list of survivors in their cohort for whom they have to collect detailed treatment data.

Data analysis of both the PanCareLIFE cohort study and the case-control study is currently under way and the first results are expected to be submitted for publication in 2019.

## Discussion

This paper describes the design and methods of 2 studies on female fertility within the PanCareLIFE project. Due to the large number of institutions collaborating within this project, these studies will encompass the largest group of CAYA cancer survivors among whom female fertility is investigated using both self-reported and hormonal data. Results will provide detailed insight into the prevalence of fertility impairment following CAYA cancer and the diagnostic- or treatment-related factors associated with an increased risk of fertility impairment. This will help clinicians to adequately counsel both girls or young women who are about to start anticancer treatments as well as adult female CAYA cancer survivors about issues concerning their remaining reproductive life span and the possible need for fertility preservation interventions. Moreover, knowledge gained from the 2 studies can be incorporated into existing evidence-based clinical guidelines on female fertility for CAYA cancer patients and survivors [28,29].

The 2 fertility studies conducted within PanCareLIFE have several strengths. First, the international collaboration, as achieved in PanCareLIFE, has resulted in an unprecedented number of female CAYA cancer survivors for whom data on fertility impairment are available. Large study populations are essential to achieve statistically and clinically meaningful results. Moreover, the large sample size in the PanCareLIFE fertility studies will allow many subgroup analyses. For these analyses, survivors whose former treatment is presumed not to negatively affect fertility (as indicated by the literature available at time of data analyses) can serve as the reference group when calculating effect measures, such as relative risks or odds ratios. Second, within both PanCareLIFE fertility studies, a broad definition of fertility impairment has been employed using several criteria that have frequently been used in previous studies assessing fertility in female CAYA cancer survivors [11,12,30-33]. By doing so, a large set of data, as provided by the data providers, who collected their data prior to the PanCareLIFE project, could be incorporated into the PanCareLIFE fertility studies. Moreover, using a broad definition of fertility impairment will enable calculation of an overall prevalence of fertility impairment and the calculation of the specific criterion-specific prevalence of fertility impairment. Each criterion-specific prevalence can then be compared with those reported in previous studies among CAYA cancer survivors, which used the same definition (ie, criterion) for fertility impairment. Third, some criteria of fertility impairment employed within PanCareLIFE included self-reported outcomes that are validated by hormonal values. Attempts to endorse questionnaire-based fertility data by comparing them with objective hormonal markers is important because it is known that self-reported fertility data, especially on menstrual cycle regularities, have limited association with objective clinical markers [34].

For the cohort study, survivors are considered *fertility impaired* when they meet at least one of 8 criteria. For the nested case-control study, however, a hierarchy is applied to these criteria, meaning that a survivor is defined as a case based on the criterion that established fertility impairment with the presumed highest level of certainty, considering the ability of this criterion to also identify “true controls” (ie, survivors who are definitely *not* fertility impaired based on that criterion by the end of follow-up). The hierarchy applied to the 8 criteria is based on several considerations. Criteria 1 through 3 (primary or secondary amenorrhea combined with a high FSH and/or a low AMH and high FSH combined with a low AMH) are deemed strong indicators of fertility impairment because one marker of (in)fertility (amenorrhea and high FSH, respectively) is validated by another marker (ie, AMH). AMH is currently considered the marker of choice when it comes to measuring ovarian reserve because it seems to be the most stable marker, it is randomly measurable throughout the menstrual cycle and it seems to reflect reduced ovarian function early in the sequence of events leading to menopause [35,36]. Criteria 4 and 5 also include the outcome amenorrhea; however, in the cases of these criteria, it is not validated by FSH or AMH values, making them less certain criteria for fertility impairment because amenorrhea may also be caused by factors other than ovarian follicle depletion [37]. Women less than 30 years of age with an AMH level below <0.5 µg/L (criterion 6) are also considered to be fertility impaired [38]. However, because hormonal contraception use has shown to significantly decrease AMH levels [25], this criterion is evaluated among the nonhormone users only. The final 2 criteria include self-reported measures regarding pregnancy attempts (use of artificial reproductive techniques and unsuccessful pregnancy attempts for at least 12 months, respectively). Both criteria have proven to be good indicators of sub- or infertility [39,40]. They are, however, at the bottom of the hierarchy because they only apply to the subgroup of survivors who have already tried to become pregnant. Consequently, these criteria do not provide any information regarding fertility impairment in those who have not yet attempted to conceive at time of study assessment, as was true for a substantial portion (approximately two-thirds) of the included survivor population. By placing criteria 1 through 6 before 7 and 8, the number of women who are categorized as being fertility impaired based on the criteria that provide information on fertility impairment in the whole cohort, and not just in those who have attempted to become pregnant, is maximized. Moreover, it enables us to easily differentiate between fertility impairment rates of survivors based on all 8 criteria versus criteria 1 through 6 only.

The fertility studies within PanCareLIFE have some limitations. First, due to missing information, some of the data on fertility impairment collected in previous local studies cannot be successfully recoded to make them compatible with the data that are collected with the PanCareLIFE questionnaire. As a consequence, data from some cohorts cannot be considered when calculating the overall prevalence of fertility impairment. Second, our studies may be subject to selection bias because from about 60% of the total invited group of subjects’ outcome data from are available for the cohort study and even less for the nested case-control study. This could impact the generalizability of our study results. To estimate the risk of selection bias, participants will be compared with nonparticipants relative to age at time of study assessment and disease-related characteristics. Third, no information is available on the fertility outcomes of women treated for CAYA cancer who died before the study (after having survived for at least 5 years). Because many of these women might have been treated with relatively high (gonado)toxic treatment regimens, they would most probably have met at least one of the 8 criteria of fertility impairment, were they still living. As a consequence, the risk of fertility impairment calculated based on this study results might be an underestimation of the “true” risk. Furthermore, for some (sub)cohorts, not all self-reported or hormonal data needed to evaluate each of the 8 criteria are available, because these data were not collected during the local fertility study in the past. As a result, survivors within certain cohorts can be evaluated by one or 2 criteria only. Hypothetically, these women could also have met one of the other criteria. However, because data for these other criteria are lacking, this group of women might be misclassified (ie, categorized as being *not fertility impaired*, whereas in reality, they are), also possibly leading to an underestimation of the overall prevalence of fertility impairment. For future studies, it is of high importance that researchers achieve consensus concerning the assessment of fertility impairment among female CAYA cancer survivors in late effects studies.

In summary, the 2 fertility studies conducted within PanCareLIFE will generate evidence-based knowledge concerning risk factors for impaired fertility among female CAYA cancer survivors as well as valuable information regarding differences in the prevalence of fertility impairment using different criteria to define this impairment. These results will enhance clinical practice because they will help health care practitioners provide adequate counseling concerning future parenthood to CAYA cancer survivors as well as new patients and refer these individuals to a reproductive specialist for fertility preservation in a timely manner. The ultimate objective is to empower patients and survivors and improve their quality of life.

## Appendix

Multimedia Appendix 1 Expanded version of Table 1 (characteristics of cohorts included in the cohort study and the nested case-control study).

Multimedia Appendix 2 Flow of data collected for the two fertility studies within PanCareLIFE project.

## Abbreviations

AMH: anti-Müllerian hormone
AUMC: Amsterdam UMC, Vrije Universiteit
CAYA: childhood, adolescent, and young adult
FSH: follicle stimulating hormone
WP: work package
