# Supplementary material for: Fertility Among Female Survivors of Childhood, Adolescent, and Young Adult Cancer: Protocol for Two Pan-European Studies (PanCareLIFE)
Source: JMIR Res Protoc. 2018 Sep 14;7(9):e10824. doi: 10.2196/10824 (PMC6231763; doi:10.2196/10824)
Supplement: Multimedia Appendix 1 [file resprot_v7i9e10824_app1.pdf]

Multimedia Appendix 1. Characteristics of cohorts included in the cohort study and the nested case-control study.

Country	Data provider or Institute	Name of study cohort	Time period of primary diagnoses	Cancer diagnosis	Type of data	Total base cohort size (n)[a]	Number of women invited (n)	Number of question-naires provided (n)	Number of serum samples provided (n)	Number of FSH levels provided (n)	Time period of data collection	Included in c-c study[b]
Netherlands	DCOG LATER[c] (Amsterdam UMC, Vrije Universiteit Amsterdam, University of Amsterdam, Erasmus Medical Center Rotterdam)	DCOG LATER cohort [17]	1963-2001	Various diagnoses	PR[d]	2190	1684	1,109	619	615	2004-2014	Yes[e]
	Netherlands Cancer Institute Amsterdam	Hodgkin Lymphoma cohort [19, 20]	1966-2000	Hodgkin Lymphoma	PR	450	275	203	0	0	1997-2016	No[f]
Germany	Universitätsklinikum Bonn	VIVE cohort	1979-2003	Various diagnoses	PR	5909	4467	2,482	0	0	2014-2015	No
	Westfaelische Wilhelms-Universitaet Muenster	Ewing 2008 Clinical Trials cohort	1999-2009	Ewing's sarcoma	DU[g]	161	140	46	24	3	2015-2016	Yes
	Charité - Universitätsmedizin Berlin	Berlin Hormone Analyses cohort [3]	1980-2007	Various diagnoses	PR	402	344	83	69	83	2008-2009	No
Czech Republic	Fakultni nemocnice Brno	Cohort female 5-y cancer survivors Brno	1977-2011	Various diagnoses	DU	283	203	182	180	83	2015-2016	Yes
	Fakultni nemocnice v Motol	Cohort female 5-y cancer survivors Motol	1973-2011	Various diagnoses	DU	1398	1063	574	301	202	2014-2016	Yes
Italy	Istituto Giannina Gaslini	Gaslini female survivors cohort	1965-2010	Various diagnoses	DU	1111	814	563	122	110	2015-2016	Yes
Switzerland	University of Bern	Swiss Childhood Cancer Survivor Study cohort 1 [18]	1976-2005	Various diagnoses	PR	1135	977	685	0	0	2007-2013	No
		Swiss Childhood Cancer Survivor Study cohort 2 [18]	2006-2010	Various diagnoses	PR	335	228	113	0	0	2015-2016	No
United Kingdom	Great Ormond Street	Hematopoietic stem	1978-2011	Various diagnose	DU	95	93	50	44	44	2015-2016	Yes

	Children’s Hospital and University College London Hospital	cell transplantation cohort		s								
Norway	Oslo University Hospital	Lymphoma survivor cohort [8]	1970-2000	Lymphoma	PR	Unknown	82	51	46	50	2007-2009	Yes
		Acute lymphoblastic leukemia survivor cohort [21]	1970-2002	Acute lymphoblastic leukemia	PR	175	103	82	65	0	2009-2010	Yes
France	University hospital Saint-Étienne	Rhone Alpe cohort 1	1987-1992	Various diagnoses	PR	212	120	120	35	13	2005-2013	Yes
		Rhone Alpe cohort 2	1993-1999	Various diagnoses	PR	284	220	102	62	0	2015-2016	Yes
Israel	Edmond and Lily Safra Children’s Hospital, Sheba Medical Center	The Edmond and Lily Safra Children’s Hospital Late Effects cohort	1981-2011	Various diagnoses	DU	239	151	102	73	36	2015-2016	Yes
Total						14,379	10,964	6547	1640	1242		

[a]Base cohort: those subjects fulfilling inclusion criteria of study.
[b]C-c study: case-control study.
[c]DCOG LATER: Dutch Childhood Oncology Group - Long term Effects after Childhood Cancer
[d]PR: data collected prior to PanCareLIFE project.
[e]Yes: institutes participating in the nested case-control study.
fNo: institutes not participating in the nested case-control study.
[g]DU: data collected during PanCareLIFE project.
