# Supplementary figures and images for: Fertility Among Female Survivors of Childhood, Adolescent, and Young Adult Cancer: Protocol for Two Pan-European Studies (PanCareLIFE)
Source: JMIR Res Protoc. 2018 Sep 14;7(9):e10824. doi: 10.2196/10824 (PMC6231763; doi:10.2196/10824)

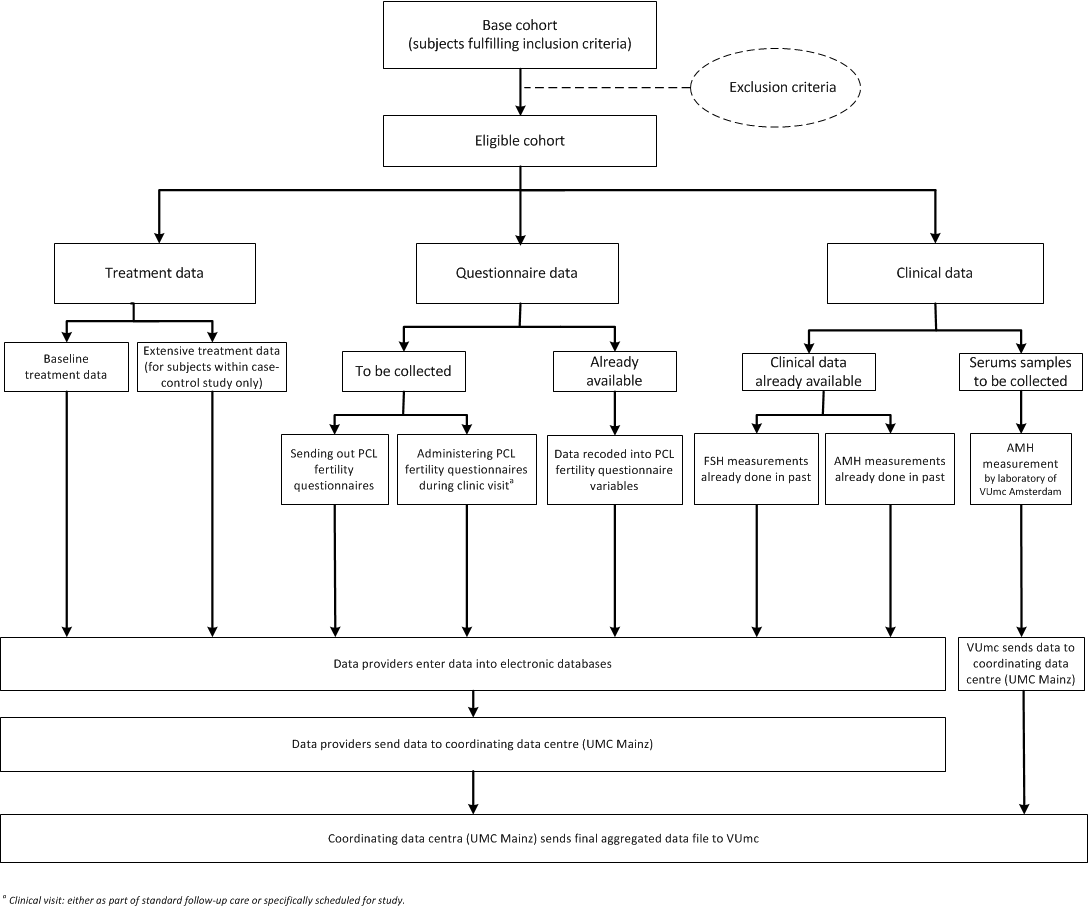

Supplement: Multimedia Appendix 2 [file resprot_v7i9e10824_app2.png]
